# CATNUS: Coordinate-Aware Thalamic Nuclei Segmentation Using T1-Weighted MRI

**Published:** 2025-12-05

**Authors:** Anqi Feng, Zhangxing Bian, Samuel W. Remedios, Savannah P. Hays, Blake E. Dewey, Alexa Colinco, Jiachen Zhuo, Dan Benjamini, Jerry L. Prince

**Affiliations:** 1 Department of Electrical and Computer Engineering, Johns Hopkins University, Baltimore, MD, USA; 2 Laboratory of Behavioral Neuroscience, National Institute on Aging, National Institutes of Health, Baltimore, MD, USA; 3 Department of Computer Science, Johns Hopkins University, Baltimore, MD, USA; 4 Department of Neurology, Johns Hopkins School of Medicine, Baltimore, MD, USA; 5 Department of Diagnostic Radiology and Nuclear Medicine, University of Maryland School of Medicine, Baltimore, MD, USA

**Keywords:** Thalamic Nuclei Segmentation, Quantitative T1 Mapping, Coordinate Convolution

## Abstract

Accurate segmentation of thalamic nuclei from magnetic resonance images is important due to the distinct roles of these nuclei in overall brain function and to their differential involvement in neurological and psychiatric disorders. However, segmentation remains challenging given the small size of many nuclei, limited intrathalamic contrast and image resolution, and inter-subject anatomical variability. In this work, we present CATNUS (Coordinate-Aware Thalamic Nuclei Segmentation), segmenting 13 thalamic nuclei (or nuclear groups) using a 3D U-Net architecture enhanced with coordinate convolution layers, which provide more precise localization of both large and small nuclei. To support broad clinical applicability, we provide pre-trained model variants that can operate on quantitative T1 maps as well as on widely used magnetization-prepared rapid gradient echo (MPRAGE) and fast gray matter acquisition T1 inversion recovery (FGATIR) sequences. We benchmarked CATNUS against established methods, including FreeSurfer, THOMAS and HIPS-THOMAS, demonstrating improved segmentation accuracy and robust test-retest reliability across multiple nuclei. Furthermore, CATNUS demonstrated strong out-of-distribution generalization on traveling-subject datasets spanning multiple scanners, field strengths, and vendors, producing reliable and anatomically coherent segmentations across diverse acquisition conditions. Overall, CATNUS provides an accurate and generalizable solution for thalamic nuclei segmentation, with strong potential to facilitate large-scale neuroimaging studies and support real-world clinical assessment.

## 1 Introduction

The thalamus is a pair of ovoid, gray matter structures located deep within the brain. It is a major component of the diencephalon, sitting above the brainstem, flanking the third ventricle, and bordered by the cerebral hemispheres [1, 2]. The thalamus not only serves as a central relay for directing sensory and motor signals to their target cortical areas, but also actively contributes to motor control, consciousness, alertness, and sleep regulation [3–6].

The thalamus consists of multiple nuclei, each connected to specific cortical and subcortical regions [1]. For example, the ventral lateral (VL) nucleus relays cerebellar signals to the motor and premotor cortex to support movement coordination, while the ventral anterior (VA) nucleus passes signals from the basal ganglia to the motor and premotor cortex to help with movement planning [7]. The ventral posterior lateral nucleus (VPL) and ventral posterior medial nucleus (VPM) process somatosensory information from the body and face, and project to the somatosensory cortex and insular cortex [7, 8]. The mediodorsal (MD) nucleus supports higher cognitive functions like working memory and emotion regulation [9], whereas the pulvinar nucleus helps control visual attention and sensory integration [10]. Additionally, disruptions in thalamic nuclei are implicated in multiple neurological and psychiatric disorders. For example, alterations in the MD nucleus are associated with schizophrenia and major depressive disorder [11]; atrophy of the anterior (AV), MD and pulvinar nuclei has been linked to cognitive impairment in Alzheimer’s disease[12]; degeneration of the centromedian (CM) nucleus contributes to motor and cognitive dysfunction in Parkinson’s disease [13]; and structural damage to the pulvinar nucleus is commonly observed in multiple sclerosis [14]. Moreover, thalamic nuclei are important targets for treatment. Deep brain stimulation (DBS) of the Vim nucleus is widely used to reduce tremors in Parkinson’s disease and essential tremor [15], while DBS targeting the anterior nucleus has been approved for the treatment of refractory epilepsy [16]. Given these diverse roles, accurate segmentation of thalamic nuclei is vital for both neuroscience research and clinical applications.

In-vivo thalamic nuclei segmentation primarily relies on Magnetic Resonance Imaging (MRI). However, the small size of nuclei and low intrathalamic contrast make manual delineation time-consuming and highly dependent on expert knowledge, even with advanced imaging techniques developed to enhance contrast [17]. These challenges highlight the need for automatic segmentation methods to achieve efficient and reliable thalamic mapping.

Recent segmentation efforts have widely adopted structural MRI (sMRI) due to its broad availability and high spatial resolution. [18] introduced an unsupervised genetic algorithm to cluster intrathalamic regions using quantitative T1 and T2 maps, though the lack of spatial constraints led to fragmented, anatomically inconsistent clusters. [19] addressed this by incorporating spatial priors into T1/T2map-derived segmentations, enabling reproducible division into six subregions. However, reliance on synthetic data with idealized contrasts, hard boundaries, and a single anatomical atlas limited its generalizability to real-world data. To improve anatomical accuracy, [20] introduced the FreeSurfer method, constructing a probabilistic atlas of 26 thalamic nuclei from ex vivo 7T MRI and histology, applied to in vivo T1-weighted Magnetization-Prepared Rapid Gradient Echo (MPRAGE) images through a Bayesian inference framework. While accurate, this approach is computationally intensive (often requiring several hours), and is prone to oversegmentation into adjacent subcortical regions. [21] proposed THOMAS, a fast multi-atlas segmentation using 7T white-matter-nulled (WMn) MPRAGE, also known as Fast Gray Matter Acquisition T1 Inversion Recovery (FGATIR), but this sequence is not commonly used in standard imaging protocols. [22] introduced a multi-contrast 7T atlas evaluated on 3T T1-weighted MRI using statistical shape models, which performed well but assumed a pre-segmented thalamic boundary and ignored intrathalamic intensity variation, limiting its sensitivity to disease-related structural changes. [23] extended THOMAS to MP2RAGE sequences, achieving comparable performance, though MP2RAGE remains less commonly used in routine clinical practice. [24] proposed a CNN-based segmentation method using standard T1-weighted MRI, with optional synthesized WMn-like contrast to enhance small nuclei detection and group difference sensitivity. Yet, it used THOMAS-derived labels as ground truth and was sensitive to the quality of synthesized contrast. Most recently, HIPS-THOMAS [25] addressed accessibility issues by generating WMn-like contrast from standard T1-weighted scans using polynomial synthesis, achieving near WMn-MPRAGE performance. However, it was evaluated by comparing against WMn-MPRAGE segmentations, rather than against manual annotations. Additionally, the simple polynomial model could amplify noise, and segmentation remained unreliable for small nuclei such as the habenula and VLa.

Diffusion MRI (dMRI) offers unique insights into thalamic microstructure by capturing local fiber orientations, enabling differentiation of nuclei beyond intensity-based sMRI. [26] performed k-means clustering on voxels within the thalamus based on the principal diffusion orientation and diffusion anisotropy. However, the method was limited by the limited angular resolution of early Diffusion Tensor Imaging (DTI), so small nuclei could not be distinguished, and it required the number of clusters to be pre-specified. [27] proposed a spectral clustering method, constructing a voxel affinity matrix based on tensor similarity and applying a normalized cut algorithm with Markov relaxation to encourage spatial coherence. Still, this method remained sensitive to parameter choices and was computationally intensive. [28] introduced a level set method, assigning voxels based on similarity to representative tensors within evolving surfaces, and incorporating fractional anisotropy (FA) and mean diffusivity (MD) to enhance segmentation. Nevertheless, the method required careful initialization, and was sensitive to noise and errors in tensor estimation. [29] computed gradients of diffusion tensor fields to highlight boundary transitions, followed by segmentation using watershed or region growing on the resulting gradient map. However, this approach was sensitive to noise and lacked priors on nuclear shape or location, leading to uncertain boundaries in regions with gradual diffusion changes. [30] clustered thalamic voxels based purely on local principal diffusion directions, assuming each nucleus has a dominant fiber orientation. While effective for large nuclei, it may merge nuclei with mixed or crossing fibers, failing to resolve small nuclei. [31] developed a multi-object geometric deformable model based on DTI, where multiple deformable surfaces (each corresponded to a nucleus) evolved simultaneously, guided by local diffusion features and shape priors, to achieve joint thalamic segmentation. However, this method required an initial thalamus mask and approximate surface initialization, and was computationally intensive. [32] proposed a k-means clustering method using orientation distribution functions (ODFs) computed from high-angular-resolution diffusion imaging (HARDI), combining ODF shape and spatial proximity to segment seven thalamic groups. While accurate, the method demanded advanced acquisition protocols and careful parameter tuning. Despite the methodological advances, dMRI-based approaches generally face several limitations. First, most acquisitions rely on echo planar imaging (EPI) [33], which provides relatively low spatial resolution (typically 2 to 3 mm isotropic), leading to partial volume effects that obscure small and adjacent nuclei. Moreover, the thalamus is primarily composed of gray matter, which exhibits low diffusion anisotropy and thus offers limited contrast for nuclei differentiation.

Beyond local microstructural features, diffusion MRI can also distinguish thalamic nuclei based on their unique patterns of structural connectivity with cortical and subcortical regions. [34] pioneered a probabilistic tractography method that parcellated the thalamus based on voxel-wise connectivity to predefined cortical regions, enabling segmentation aligned with functional cortico-thalamic circuits. However, the method depended on arbitrary cortical region-of-interest (ROI) definitions and might not align with true anatomical boundaries. [35] validated this approach by demonstrating correspondence between connectivity-defined segments and both histological nuclei and functional MRI (fMRI) activations. However, this validation did not address the method’s original limitations, and the approach was not evaluated for small nuclei. [36] applied Independent Component Analysis (ICA) to thalamocortical tractography data to identify independent connectivity patterns, enabling thalamic parcellation without predefined cortical ROIs. Still, it did not guarantee one-to-one alignment with traditional nuclei, often spanning multiple cytoarchitectonic nuclei or splitting one nucleus into pieces with different connectivity. Overall, connectivity-based thalamic nuclei segmentation faces several limitations. First, it often fails to match the true anatomical boundaries of thalamic nuclei. This is partly because many neighboring nuclei project to similar cortical regions, leading multiple distinct nuclei to be grouped together. Also, some individual nuclei exhibit heterogeneous projection patterns, which can lead to a single anatomical nucleus being split into multiple fragments. Moreover, crossing or complex fibers make tractography results less reliable. In such regions, the inferred pathways can be inaccurate or incomplete, which leads to incorrect connectivity assignments for thalamic voxels.

To better leverage the complementary strengths of structural and diffusion MRI, several studies have explored hybrid approaches for thalamic nuclei segmentation. [37] introduced a hierarchical random forest that first segmented the whole thalamus and then parcellated it into six nuclei, using T1-weighted MRI, diffusion-derived features, and tractography-based connectivity. [38] extended this method by incorporating atlas-derived nuclei priors to better localize small structures like the lateral and medial geniculates. However, both approaches shared limitations, such as dependence on accurate whole-thalamus segmentation, limited generalizability due to small training sets, and sensitivity to tractography quality, especially in low-resolution or noisy diffusion data. [39] extended the MPRAGE-based FreeSurfer method by introducing a Bayesian framework that integrates sMRI and dMRI through joint likelihood modeling, combining an improved probabilistic atlas as prior to estimate voxel-wise posterior probabilities, with final labels assigned via MAP estimation. Yet, the method did not model partial volume effects and imposed a reflective symmetry constraint on dMRI distributions, which might limit accuracy in low-resolution data or anatomically asymmetric cases. [40] adopted Uniform Manifold Approximation and Projection (UMAP) to project multimodal MRI features, including T1-weighted, T2-weighted, multiple T1-weighted images at different inversion times (multi-TI) and diffusion-derived features, into a low-dimensional space, and performed thalamic nuclei segmentation using k-nearest neighbor classification. [41] and [42] both adopted a two-stage 3D U-Net architecture that first segmented the whole thalamus and then further parcellated the thalamus into 13 nuclei, leveraging multimodal data including multi-TI images, quantitative T1 and proton density (PD) maps and diffusion-derived features. Despite these efforts, the use of multimodal features is still subject to several practical constraints. First, different modalities capture distinct structural properties: sMRI provides anatomical boundaries, dMRI encodes local microstructural information, and tractography-derived connectivity reflects long-range projections. These representations often misalign spatially or functionally, leading to conflicting cues at nucleus boundaries, where anatomically distinct nuclei may share similar connectivity profiles, and a single nucleus may show heterogeneous diffusion patterns. Second, most current multimodal methods rely on supervised learning, yet the available ground truth labels are typically derived from sMRI or dMRI alone and may not correspond well across modalities, reducing supervision reliability when conflicts arise. Moreover, the resolution gap between high-resolution sMRI and typically lower-resolution dMRI limits the visibility of small nuclei in diffusion space, further complicating voxel-level fusion across modalities.

In this work, we present CATNUS (Coordinate-Aware Thalamic Nuclei Segmentation), a deep learning-based framework for thalamic nuclei segmentation built upon a coordinate-aware 3D U-Net [43], targeting 13 nuclei (or nuclear groups). CATNUS accepts quantitative T1 maps as primary input, which offer favorable contrast for thalamic nuclei segmentation [44], and can also operate on MPRAGE or FGATIR for broader clinical applicability. This work extends our earlier studies [42, 44] by incorporating coordinate convolution, expanding benchmark comparisons, and performing out-of-distribution (OOD) tests using traveling-subject datasets. The main contributions are threefold: (1) CATNUS achieves accurate segmentation of all 13 thalamic nuclei, including small anatomically challenging structures, by integrating coordinate convolution [45] to improve spatial encoding; (2) it outperforms existing methods such as FreeSurfer, THOMAS, and HIPS-THOMAS, demonstrating improved performance and robust test-retest reliability across multiple nuclei; (3) it shows strong generalization to out-of-distribution traveling-subject datasets, producing anatomically consistent segmentations across different scanners and imaging conditions. Overall, CATNUS provides an accurate and generalizable framework for thalamic nuclei segmentation, with strong potential for both research and clinical use.

## 2 Methods

### 2.1 Overview

This section outlines the overall methodological workflow of CATNUS, including data preparation, model design, and evaluation alignment. Section 2.2 introduces the MRI acquisition protocol and imaging parameters. Section 2.3 details the preprocessing, quantitative mapping, and multi-TI computation pipeline. Section 2.4 describes the manual delineation procedure. Section 2.5 presents the coordinate-aware 3D U-Net segmentation framework, including network architecture, data augmentation, loss function, and post-processing refinement. Finally, Section 2.6 explains the unified labeling protocol used to align our nuclei definitions with those of existing methods for comparison.

### 2.2 Data Acquisition

The MRI dataset used for the development and primary evaluation of CATNUS was acquired as part of a study on a mild traumatic brain injury (mTBI), approved by the University of Maryland’s ethics review board. The dataset includes 24 participants (14 healthy controls and 10 individuals with mTBI) for model training and evaluation, and an additional 10 healthy subjects with two MRI sessions acquired six months apart for the test–retest analysis. Each participant underwent an MRI session with MPRAGE and FGATIR sequences, both acquired at 1 mm isotropic resolution with a repetition time (TR) of 4000 ms, an echo time (TE) of 3.37 ms, and a flip angle of 6°. The two sequences differed only in the inversion time (TI): 1400 ms for MPRAGE and 400 ms for FGATIR. All scans were performed on a 3T SIEMENS Prisma scanner.

### 2.3 Data Preprocessing and Computation

**Figure 1:**
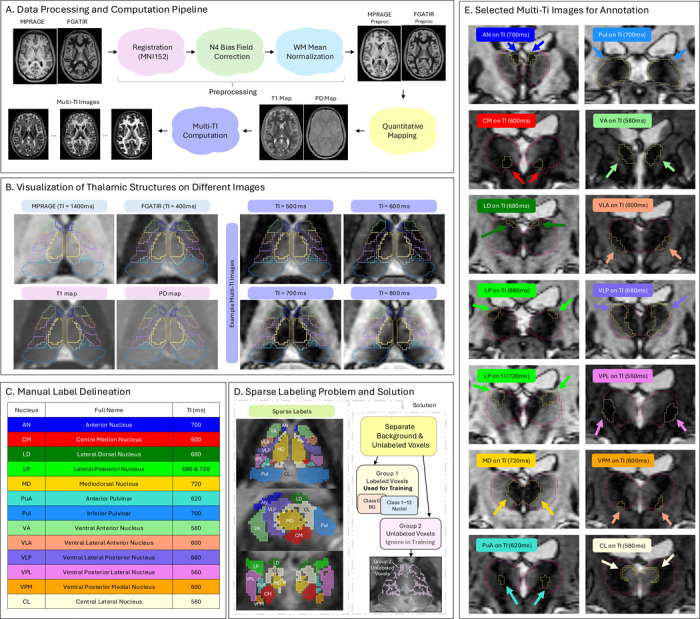
Overview of the CATNUS preprocessing and annotation workflow. A. Data preprocessing and computation pipeline. MPRAGE and FGATIR images are co-registered to the MNI152 space, followed by joint N4 bias field correction and white matter (WM) mean normalization. Quantitative T1 and PD maps are computed, and multi-TI images are generated using the estimated T1 and PD maps. B. Visualization of thalamic structures on different images. Representative MPRAGE, FGATIR, T1 and PD maps, and multi-TI images illustrate how varying inversion times enhance intra-thalamic visibility and facilitate nuclear delineation. C. Manual label delineation. Summary of the 13 thalamic nuclei with their abbreviations, full names, selected TI values used for annotation, and the color coding scheme. D. Sparse-labeling problem and solution. Annotations were restricted to high-confidence voxels (left). Unlabeled voxels were explicitly separated from background and excluded from model training (right). E. Selected multi-TI images for annotation. Examples of nuclei annotated on specific TI images, illustrating how different inversion times highlight distinct nuclear boundaries. Red and yellow outlines indicate the thalamic boundary and the corresponding nucleus boundary, respectively. When only yellow is visible, the nucleus boundary coincides with the thalamic boundary on this slice.

We processed MPRAGE and FGATIR images to compute quantitative T1 and PD maps, which were then used to calculate multi-TI images. The T1 maps served as inputs for segmentation models, while the multi-TI images were used for thalamic nuclei delineation and visualization. The preprocessing and computation pipeline is described in detail below and illustrated in Figure 1A. Example MPRAGE and FGATIR images, T1 and PD maps, and selected multi-TI images are shown in Figure 1B.

We selected T1 maps as the primary input modality because they encodethecoretissue-specificT1contrastthatunderliesT1-weighted acquisitions. As demonstrated in our previous work [44], T1 maps provide better segmentation performance compared to individual T1-weighted sequences such as MPRAGE or FGATIR. However, not all clinical environments can acquire the dual inversion time scans needed to compute T1 maps. Thus, we additionally trained models on MPRAGE and FGATIR separately to maximize clinical accessibility.

#### Data Preprocessing

Image preprocessing consisted of three sequential steps: (1) Registration. MPRAGE and FGATIR images were co-registered to the MNI152 atlas using a coarse-to-fine strategy with ANTs [46], where MPRAGE was first rigidly aligned to the MNI template, followed by FGATIR alignment to MPRAGE. (2) N4 bias field correction using N4ITK [47]. (3) White matter mean normalization [48]. Because MPRAGE and FGATIR were acquired with identical parameters except for the inversion time (TI), their quantitative relationship must be carefully preserved for reliable signal modeling and parameter estimation. Therefore, these steps were applied jointly to ensure that both modalities remain on a consistent intensity scale.

##### N4 bias field correction.

Let $I_{\text{M}}(x)$ and $I_{\text{F}}(x)$ denote the MPRAGE and FGATIR images after registration to the MNI space. We first estimated the bias fields independently for each modality using N4:

(1) $$B_{\text{M}}(x)=\text{N}4\left(I_{\text{M}}(x)\right),\;B_{\text{F}}(x)=\text{N}4\left(I_{\text{F}}(x)\right).$$

To harmonize correction across modalities, we computed the geometric mean of the two bias fields:

(2) $$B_{\text{harm}}(x)=\sqrt{B_{\text{M}}(x)\cdot B_{\text{F}}(x)}.$$

This harmonized bias field was then applied to both images:

(3) $${\hat{I}}_{\text{M}}(x)=\frac{I_{\text{M}}(x)}{B_{\text{harm}}(x)},\;{\hat{I}}_{\text{F}}(x)=\frac{I_{\text{F}}(x)}{B_{\text{harm}}(x)}.$$

##### White matter mean normalization.

We generated a white matter (WM) mask $M_{\text{WM}}$ from the MPRAGE image using fuzzy C-means clustering, and computed the mean WM intensity:

(4) $$\mu_{\text{WM}}=\frac{1}{\left|M_{\text{WM}}\right|}\sum_{x\in M_{\text{WM}}}{\hat{I}}_{\text{M}}(x),$$

where $\left|M_{\text{WM}}\right|$ is the total number of voxels in the white matter mask. Both bias-corrected images were then scaled by this mean value:

(5) $${\tilde{I}}_{\text{M}}(x)=\frac{{\hat{I}}_{\text{M}}(x)}{\mu_{\text{WM}}},\;{\tilde{I}}_{\text{F}}(x)=\frac{{\hat{I}}_{\text{F}}(x)}{\mu_{\text{WM}}}.$$

With these preprocessing steps, the images are standardized for accurate quantitative estimation.

#### Quantitative Mapping and Image Computation

For a given voxel v within a T1-weighted inversion recovery image, the signal intensity I(v) can be modeled as:

(6) $$I(v)=PD(v)\left[1-2\exp\left(-\frac{\text{TI}}{T_{1}(v)}\right)+\exp\left(-\frac{\text{TR}}{T_{1}(v)}\right)\right],$$

where PD(v) and $T_{1}(v)$ denote the proton density and T1 relaxation value at voxel v, while TI and TR are the inversion and repetition times. Given two input images (MPRAGE and FGATIR) and their known acquisition parameters, we obtained two measurements per voxel, and fitted two instances of Equation 6 to estimate PD(v) and $T_{1}(v)$ at each voxel using nonlinear least squares fitting.

FGATIR was acquired with a short inversion time (TI = 400 ms), placing many tissues in a state of negative longitudinal magnetization. However, MR scanners store magnitude images, which discard signal polarity by taking the absolute value. To preserve the signed signal characteristics required for accurate fitting of Equation 6, we negated the FGATIR magnitude image prior to parameter estimation, effectively recovering the true signal distribution expected by the model. Note that this negation assumption holds for thalamic tissues with relatively long T1 values, but may not apply to tissues with much shorter T1 (e.g., fat).

After estimating PD(v) and $T_{1}(v)$, we computed multi-TI images to support the delineation of thalamic nuclei. Specifically, for each voxel, we used Equation 6 to calculate signal intensity by fixing TR and plugging in the estimated PD(v) and $T_{1}(v)$ values, while varying the TI. We took the absolute value of the simulated signal to match the magnitude format of clinical MR images. These multi-TI images enhance thalamic contrast by modulating tissue visibility across different inversion times. Depending on the TI, specific tissues are nulled and inter-compartment differences amplified, enabling visual identification of some thalamic nuclei boundaries that are not discernible in MPRAGE or FGATIR images. Example multi-TI images in Figure 1B show how TI variations affect tissue contrast and highlight different features. In our implementation, TI is sampled from 400 ms to 1400 ms in 20 ms increments. This range spans meaningful contrast variation across thalamic tissue, and the 20 ms step is the minimum visually detectable change in our images.

### 2.4 Manual Label Delineation

Manual delineation of thalamic nuclei was guided by the Morel Atlas [49], focusing on 13 nuclei (or nuclear groups). Multi-TI images were adopted for annotation because they provide enhanced contrast for these small, densely packed thalamic structures. For each nucleus, the rater first selected the TI image with the most distinct boundary appearance for annotation, and then verified anatomical alignment using the corresponding T1 map. Figure 1C summarizes the nuclei names, abbreviations, selected TI values for annotation, and the color-coding scheme, while Figure 1E displays the chosen TI images that guided delineation for each nucleus.

Due to the small size and limited contrast between thalamic nuclei, manual annotations were restricted to voxels for which the rater had high confidence. Thus, many voxels, particularly those near thalamic boundaries, remained unlabeled, as shown in Figure 1D (left). Labeled nuclei were assigned class indices 1–13, while both background and unlabeled voxels were initially grouped under class 0. To better distinguish true background from uncertain regions within the thalamic boundary, we manually localized the thalamus using complementary contrast information from multi-TI images. Voxels outside this region were defined as background, kept as class 0, and included in model training. Unlabeled voxels within the region were assigned a separate value (100) and excluded from model training, as shown in Figure 1D (right). This separation allowed the model to learn effectively from labeled nuclei while minimizing the influence of uncertain areas.

### 2.5 Segmentation Framework

**Figure 2:**
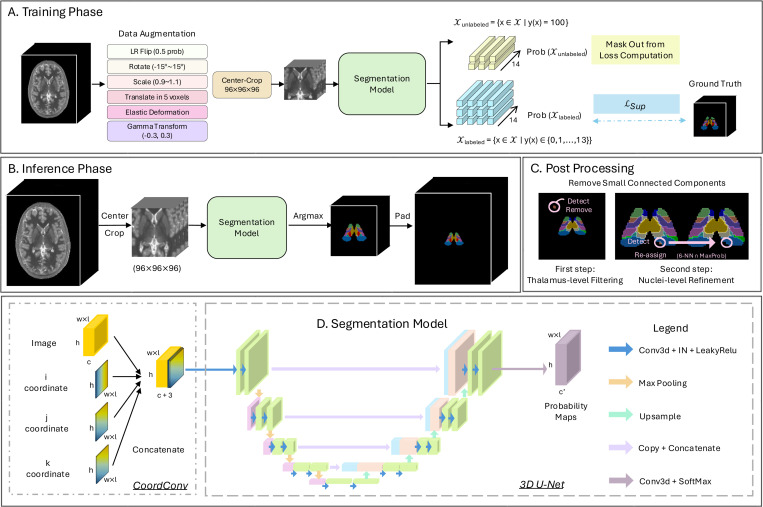
Overview of the CATNUS segmentation framework. A. Training Phase. Input images undergo spatial and intensity augmentations, followed by center-cropping to a fixed size. The model is supervised using Dice loss computed only on voxels with manual labels, while unlabeled voxels are masked out from loss computation. B. Inference Phase. A cropped image is passed through the model, and each voxel is assigned the class with the highest predicted probability. The output is then padded back to the original volume size. C. Post Processing. Predicted segmentations are refined sequentially, first by thalamus-level filtering, then by nucleus-level refinement. D. Segmentation Model. The model takes an image and its voxel-wise spatial coordinates (x, y, z) as input, processes them via coordinate convolution, and outputs probability maps using a 4-level 3D U-Net.

CATNUS employs a 3D U-Net [43] based segmentation framework to classify 14 thalamic classes (0: background, 1–13: nuclei) from single-channel input images. The model supports quantitative T1 maps, MPRAGE, or FGATIR as input. To enhance spatial encoding for small nuclei, CATNUS incorporates coordinate convolution [45] by concatenating normalized voxel coordinates (x, y, z) to the input image. The model was trained using Dice loss computed exclusively on manually labeled voxels, while unlabeled regions were excluded from loss computation. The overall workflow is illustrated in Figure 2, and the subsequent subsections detail the data augmentation, model architecture, loss formulations, and post-processing.

#### Data Augmentation

To improve model generalization and account for anatomical variability, we apply diverse data augmentation during training (illustrated in Figure 2A). Spatial augmentations include left-right flipping (p=0.5), affine transformations with rotations up to ± 15°, scaling between 0.9–1.1, and translations within 5 voxels. We also apply elastic deformation usinga 3D B-spline grid with seven control points per axis and maximum displacement of seven voxels. Following augmentation, all volumes are center-cropped to a fixed size of 96×96×96, which fully covers the thalamus region across subjects in MNI space. Spatial transformations are applied jointly to images and labels using trilinear interpolation for images and nearest-neighbor for labels. Intensity augmentation is applied only to images via random gamma correction, with log gamma values sampled uniformly from [−0.3, 0.3]. During validation and testing, only center cropping to 96×96×96 is applied, with no random transformation.

#### Model Architecture

Our segmentation model (illustrated in Figure 2D) uses a 4-level 3D U-Net [43] as the backbone, which has an encoder–decoder architecture with skip connections and a central bottleneck block.

##### Encoder Path:

The encoder contains four downsampling stages. Each stage applies two consecutive 3D convolution blocks (3×3×3 kernels, instance normalization, LeakyReLU activation), followed by 2×2×2 max pooling to reduce spatial resolution while increasing feature channels.

##### Decoder Path:

The decoder contains four upsampling stages that mirror the encoder. Each upsampling stage follows a three-step process: (1) trilinear interpolation upsampling with 2× scale factor, (2) 3D convolution with instance normalization and LeakyReLU activation, and (3) concatenation with corresponding encoder features via skip connections, followed by two 3D convolution blocks for feature integration.

##### Coordinate Convolution:

To enhance spatial awareness, particularly for small nuclei like the PuA, we incorporate coordinate convolution [45] at the input layer. This adds three channels representing normalized voxel-wise x, y, and z coordinates to the input image. The coordinates are computed for the center-cropped 96×96×96 volume after data augmentation and normalized to the range [−1, 1], with 0 corresponding to the spatial center along each axis.

##### Padding Strategy:

We replace the default zero padding in all convolutional layers with replicate padding. In coordinate convolution, the value 0 represents the spatial center along each axis. If zero padding is used, voxels with this same value would be introduced at the image borders, creating ambiguity as the network may confuse padded border regions with true center locations. Replicate padding avoids this by copying edge values, preserving the correct spatial context at the boundaries.

##### Output:

The network outputs 14-channel voxel-wise probability maps through a final 3D convolution followed by softmax activation.

#### Loss Function

We employ supervised Dice loss computed only on labeled voxels to handle the sparse annotation problem (illustrated in Figure 2A). Let the labeled voxel set be:

$$X_{\text{labeled}}=\{x\in X|y(x)\in \{0,1,\ldots ,13\}\},$$

where y(x) denotes the ground-truth label for voxel x, and X is the set of all voxels in the volume. For $x\in X_{\text{labeled}}$, we compute the mean class-wise Dice loss between the predicted probabilities $P_{\text{pred}}(x)$ and the one-hot ground-truth $P_{\text{gt}}(x)$:

$$L_{\text{dice}}=1-\frac{1}{\left|C\right|}\sum_{c\in C}\frac{2\sum_{x\in X_{\text{labeled}}}P_{\text{pred}}(c,x)\cdot P_{\text{gt}}(c,x)}{\sum_{x\in X_{\text{labeled}}}\left[P_{\text{pred}}(c,x)+P_{\text{gt}}(c,x)\right]+\varepsilon },$$

where C={0,1,…,13} includes 14 classes, with 0 as background and the rest corresponding to thalamic nuclei, and ϵ is a small constant for numerical stability. This formulation encourages overlap between the predicted and ground-truth regions for each class, while ignoring unlabeled voxels that might introduce training noise.

#### Post-processing

To improve anatomical plausibility and reduce spurious predictions, we apply two sequential post-processing steps after inference (illustrated in Figure 2C). First, to address false positive predictions in non-thalamic regions, we perform thalamus-level filtering. We create a binary mask by combining all predicted thalamic nuclei (labels 1–13) versus background (label 0), then remove any connected components that are smaller than 200 voxels, as these typically represent spurious predictions far from the main thalamic region. Second, to eliminate fragmented predictions within each class, we apply nucleus level refinement. For each predicted class, we remove disconnected components smaller than 10 voxels and reassign these isolated regions to the class with the highest predicted probability among their 6-connected neighbors. These two steps work together to promote spatial consistency in the final segmentation.

### 2.6 Labeling Schemes Unifying Protocol

**Figure 3:**
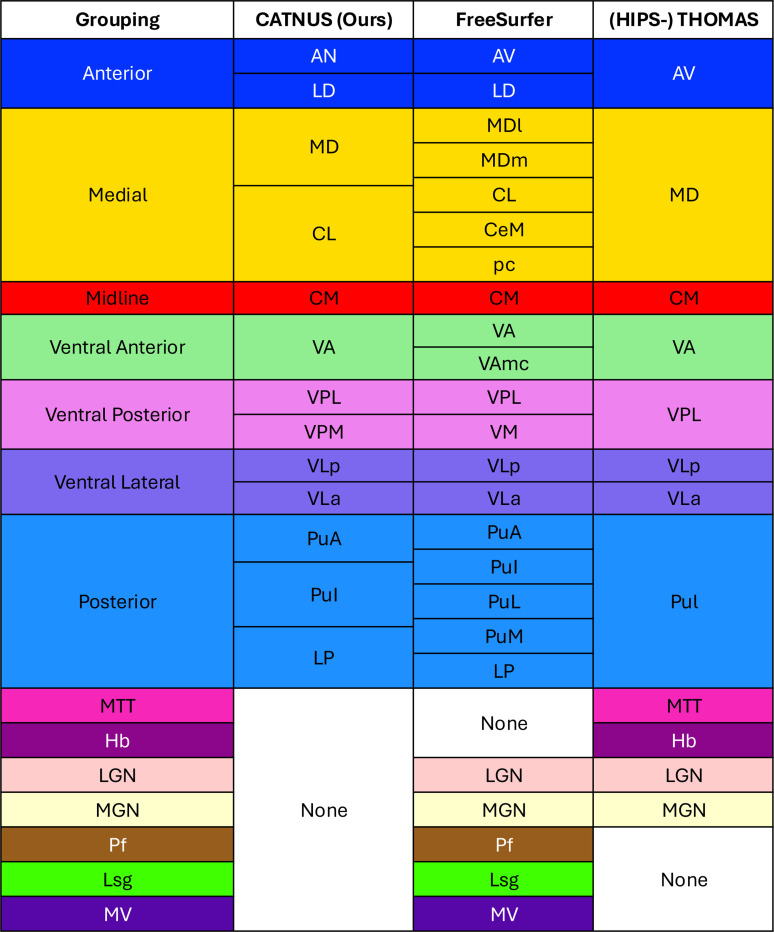
A comprehensive mapping of thalamic nuclei categories between CATNUS and the definitions from FreeSurfer, THOMAS and HIPS-THOMAS. The first column shows the major categories derived from our unified grouping strategy, each corresponding to merged nuclei listed in the second, third, and fourth columns. All major categories and individual nuclei are assigned distinct colors to help with visual differentiation. Note that THOMAS and HIPS-THOMAS share the same labeling and color conventions. Abbreviations for nuclei in FreeSurfer, THOMAS, and HIPS-THOMAS are adopted from [20], [21] and [25].

We compared CATNUS against three widely used thalamic nuclei segmentation tools: FreeSurfer, THOMAS, and HIPS-THOMAS. FreeSurfer uses a Bayesian inference framework and operates on standard MPRAGE scans [20]. THOMAS is a multi-atlas segmentation method designed for white-matter-nulled (WMn) MPRAGE [21]. Although THOMAS can also be applied to MPRAGE, its performance is substantially lower than with FGATIR. Because FGATIR is available in our dataset and provides equivalent WMn contrast, we evaluated THOMAS only on FGATIR scans. HIPS-THOMAS extends THOMAS by synthesizing WMn-like contrast from conventional T1-weighted images to improve accessibility [25]. For evaluation completeness, HIPS-THOMAS was applied to both MPRAGE and FGATIR scans. As each method follows a different labeling protocol, we used the Morel atlas [49] as a structural reference to establish a unified labeling scheme for comparison. Based on anatomical correspondences, we grouped nuclei into seven major groups: Anterior, Medial, Midline, Ventral Anterior, Ventral Posterior, Ventral Lateral, and Posterior. Nuclei without clear correspondence, such as Pf, Lsg, and MV (present only in FreeSurfer); MTT and Hb (present in THOMAS and HIPS-THOMAS); and MGN and LGN (absent from our labels), were retained as distinct classes. The grouping and color-coding scheme is illustrated in Figure 3. Notably, THOMAS and HIPS-THOMAS share the same labeling definitions, so we present them as a single column.

## 3 Experiments and Results

### 3.1 Experimental Setup

#### Experiments Overview:

We evaluated CATNUS through three main experiments: (1) within-domain segmentation across input modalities and with/without coordinate convolution (Section 3.2); (2) comparisons against established tools (Section 3.3); and (3) out-of-distribution generalization on traveling-subject datasets (Section 3.4). These experiments together assess segmentation accuracy, robustness and generalizability.

#### General Training Configuration:

Models were trained on single-channel volumetric data, where the input was either a T1 map, MPRAGE, or FGATIR. We used the Adam optimizer with an initial learning rate of 1e–3 and a weight decay of 1e–4. The batch size was set to 1 due to GPU memory constraints with 3D volumetric inputs. To ensure reproducibility and fair comparisons, we fixed the random seed to 1234.

#### Cross-validation Experiments:

For experiments requiring ground truth evaluation, we performed 8-fold cross-validation using our 24 manually labeled subjects. In each fold, 19 subjects were used for training, 2 for validation, and 3 for testing, ensuring each subject appeared in the test set exactly once. The learning rate was reduced by 10% if the validation loss did not improve for 5 consecutive epochs. Training was run for up to 200 epochs, with early stopping applied if no improvement was observed over 15 consecutive epochs. These cross-validation models were used to evaluate different input modalities and with/without coordinate convolution (Section 3.2), and to compare against benchmark methods (Section 3.3), with final performance reported as mean and standard deviation across all 24 test subjects. Because our ground truth labels are sparse, where the rater only annotated voxels with high confidence, many thalamic voxels remain unlabeled. In such cases, overlap-based metrics like the Dice score can be misleading, as they may penalize predictions that correctly identify a nucleus class but lack corresponding ground truth labels. To avoid this issue, we adopted the true positive rate (TPR) as our primary evaluation metric, which better reflects performance under partial supervision.

#### Full-dataset-training Experiment:

For out-of-distribution evaluation, we trained CATNUS variants with all 24 labeled subjects to maximize model performance. Without a validation set, the learning rate was scheduled to decay by 10% every 10 epochs, and training was run for exactly 200 epochs without early stopping. The resulting models were then used for generalization testing on external datasets (Section 3.4).

### 3.2 Segmentation Performance Across Modalities and Coordinate Convolution

#### Experimental Design:

We systematically evaluated the effects of input modality and coordinate convolution (CoordConv) on segmentation performance. Specifically, we trained six models, where the input was either a T1 map, MPRAGE, or FGATIR scan, each with or without CoordConv. All models shared the same architecture and training protocol to ensure controlled comparison. To isolate the effects of modality and coordinate encoding, no post-processing was applied during this evaluation. Performance was measured using the true positive rate (TPR) for each of the 13 thalamic nuclei. Additionally, we computed a volume-weighted average (VWA) TPR per subject to assess overall segmentation accuracy. Final results are reported as the mean and standard deviation of per-nucleus and VWA TPRs across all 24 subjects.

#### Quantitative Results:

**Figure 4:**
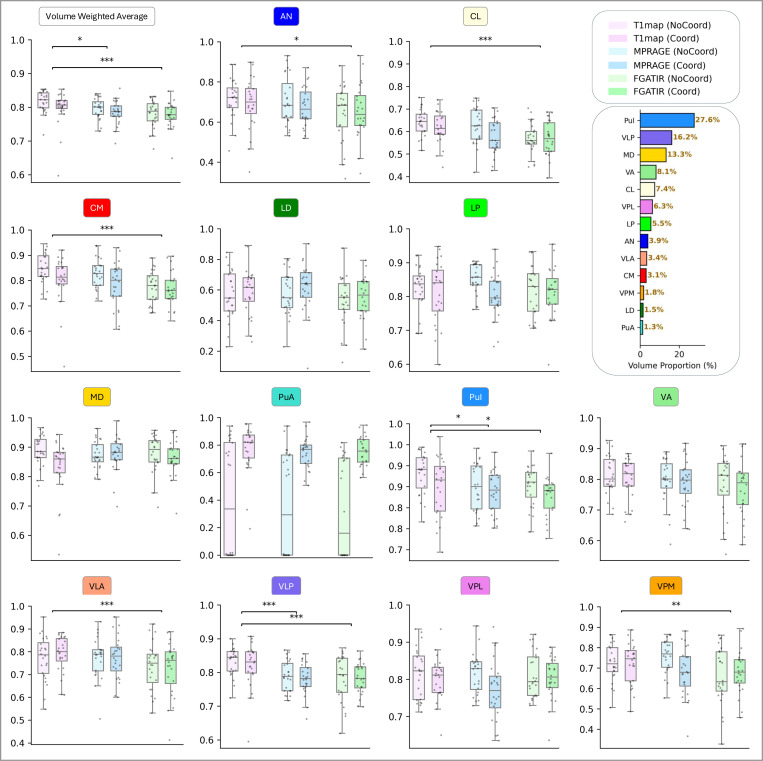
Within-domain segmentation performance across three input modalities (T1 map, MPRAGE, and FGATIR) with or without coordinate convolution (CoordConv). Each boxplot shows the distribution of true positive rates (TPR) for one nucleus or the volume-weighted average (VWA), grouped by modality and CoordConv configuration. Performance was evaluated across 24 manually labeled subjects using 8-fold cross-validation. Statistically significant differences relative to T1map (Coord) are indicated by stars (*p<0.05, **p<0.01, ***p<0.001). The top-right legend indicates color mapping for the six configurations. The horizontal bar summarizes the volume proportions of each nucleus.

Figure 4 presents the quantitative results as boxplots, where each subplot corresponds to one thalamic nucleus (or VWA) and illustrates the distribution of TPRs across all subjects for the six models. Overall, models trained on T1 maps achieved higher TPRs than those using MPRAGE or FGATIR for most nuclei and in the VWA metric. Coordinate convolution did not consistently improve performance across all regions. However, for the PuA nucleus, which is the smallest structure among all nuclei, the absence of CoordConv often resulted in poor or failed predictions. In contrast, including coordinate information significantly enhanced performance. These findings highlight the importance of explicit spatial encoding, particularly for segmenting small and anatomically subtle nuclei like PuA.

#### Statistical Analysis:

We first conducted a two-way repeated measures ANOVA. The analysis was performed separately for each of the 13 thalamic nuclei and the VWA TPRs. Input modality and CoordConv configuration were treated as within-subject factors, with TPR as the dependent variable. To correct for potential violations of sphericity, Greenhouse–Geisser correction was applied, and generalized eta-squared $\eta^{2}$ was reported as the effect size. The ANOVA revealed a significant main effect of modality in 8 nuclei, specifically AN, CL, CM, LD, Pul, VLP, VLA, VPM, as well as in the VWA, indicating that segmentation performance varied systematically across input modalities. However, no significant main effect of CoordConv or a significant interaction between modality and CoordConv was detected, suggesting that coordinate convolution did not consistently influence segmentation accuracy. Given the significant effects of input modality, we further conducted post-hoc pairwise Wilcoxon signed-rank tests between all modality pairs (T1 map vs. MPRAGE, T1 map vs. FGATIR, MPRAGE vs. FGATIR), with Bonferroni correction for multiple comparisons. Since CoordConv showed no significant effect, results from models with and without CoordConv were pooled to increase statistical power without introducing confounding factors. The results are visualized in Figure 4, with statistically significant differences marked by asterisks (*p<0.05, **p<0.01, ***p<0.001). For VWA, which reflects an overall segmentation performance, T1 map significantly outperformed FGATIR and MPRAGE. At the individual nucleus level, T1 map achieved significantly higher TPRs than FGATIR in seven nuclei (AN, CL, CM, PuI, VLA, VLP, VPM) and than MPRAGE in two nuclei (PuI, VLP). No significant differences between modalities were observed in LD, LP, MD, PuA, VA, or VPL.

#### Qualitative Results:

**Figure 5:**
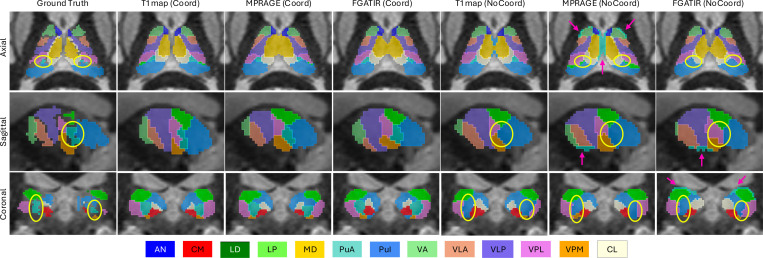
Qualitative comparisons of segmentation results across input modalities (T1 map, MPRAGE, FGATIR) and coordinate convolution (CoordConv) settings, overlaid on FGATIR images. Each row shows a representative slice in a different anatomical plane (axial, sagittal, coronal), with each slice taken from a different subject. Ground-truth annotations (leftmost column) are compared against model predictions from the six configurations. Yellow circles highlight common failure cases, especially for the small PuA nucleus, which is frequently missed when CoordConv is not used. Pink arrows indicate additional mislocalizations of the PuA nucleus along thalamic boundaries.

Figure 5 shows segmentation outputs across input modalities and CoordConv settings. Overall, all models produced segmentations that broadly align with the ground-truth labels. Thalamic boundaries are generally accurate and smooth across all views, and most nuclei appear at their expected anatomical locations. However, notable differences are apparent for the smallest nucleus, PuA. Models without CoordConv often fail to predict PuA at the correct positions, as highlighted with yellow circles, where the PuA is visible in the ground-truth but absent in predictions from models lacking coordinate encoding. In addition, we observe systematic mislocalizations of PuA in the MPRAGE and FGATIR models without CoordConv, where false positives appear along the outer boundaries of the thalamus, indicated by pink arrows. These qualitative patterns align with the quantitative findings, reinforcing that explicit spatial encoding via CoordConv enhances anatomical accuracy and localization precision for small nuclei like PuA.

### 3.3 Comparisons Against Established Methods

#### Experimental Design:

**Figure 6:**
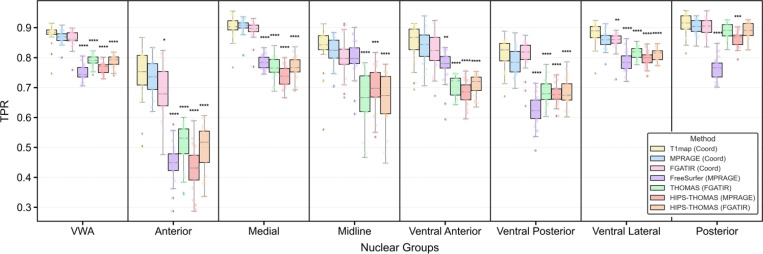
Quantitative comparison of thalamic nuclei segmentation performance against benchmark methods. Results show true positive rates (TPRs) averaged over 24 subjects from 8-fold cross-validation, evaluated for seven major nuclear groups and their volume-weighted average (VWA). The first three methods, T1map (Coord), MPRAGE (Coord), FGATIR (Coord), are our coordinate-aware CATNUS variants. The latter four are literature baselines: FreeSurfer applied to MPRAGE scans, THOMAS applied to FGATIR scans, and HIPS-THOMAS applied to either MPRAGE or FGATIR images. Each box shows the distribution of TPRs for a given nuclear group and method, with overlaid dots indicating subject-level values. Asterisks indicate statistically significant differences from T1map (Coord) after Bonferroni correction (*p<0.05, **p<0.01, ***p<0.001, ****p<0.0001).

We benchmarked our three coordinate-aware models (trained separately on T1 map, MPRAGE, and FGATIR) against three established segmentation methods, FreeSurfer [20], THOMAS [21] and HIPS-THOMAS [25]. As described in Section 2.6, we ran FreeSurfer with MPRAGE, THOMAS with FGATIR, and HIPS-THOMAS with either MPRAGE or FGATIR. All input images underwent the identical preprocessing pipeline described in Section 2.3, including registration to MNI152 template, N4 bias field correction, and white matter mean normalization. Our model predictions, the benchmark segmentation results, and the ground-truth labels were mapped to the unified scheme depicted in Figure 3 for comparisons. For quantitative analysis, we focused only on the seven common nuclear groups, excluding the background and nuclei unique to a single method. We computed the true positive rate (TPR) for each of the seven groups by comparing model outputs with the ground truth, and derived a volume-weighted average (VWA) TPR across groups. For each method, we reported the mean value and standard deviation of these per-subject metrics and visualized the full distributions in Figure 6. Statistical comparisons were performed using paired Wilcoxon signed-rank tests against our coordinate-aware model trained with T1 maps, selected for its strong overall performance. Bonferroni correction was applied for multiple comparisons, and significance levels are indicated as follows: *p<0.05, **p<0.01, ***p<0.001, ****p<0.0001.

#### Quantitative Results:

In Figure 6, all three coordinate-aware models substantially outperformed the benchmark methods, both in volume-weighted average (VWA) and across nuclear groups. Among the three variants, the T1 map with CoordConv model achieves the best performance, reaching a VWA TPR of 0.88 ± 0.03, significantly higher than FreeSurfer (0.75 ± 0.03; p < 0.0001), THOMAS (0.79 ± 0.02; p < 0.0001), and HIPS-THOMAS on MPRAGE (0.76 ± 0.02; p < 0.0001) and FGATIR (0.79 ± 0.02; p < 0.0001). At the nuclear group level, it shows significant improvements over FreeSurfer in the Anterior, Medial, Ventral Anterior, Ventral Posterior, Ventral Lateral, and Posterior groups, and over THOMAS in the Anterior, Medial, Midline, Ventral Anterior, Ventral Posterior, and Ventral Lateral groups. Further, it outperforms HIPS-THOMAS (MPRAGE) in all groups and HIPS-THOMAS (FGATIR) in the Anterior, Medial, Midline, Ventral Anterior, Ventral Posterior, and Ventral Lateral groups. The T1 map with CoordConv model also displays a consistent trend of higher TPRs than its MPRAGE and FGATIR counterparts, though most differences are not statistically significant.

#### Qualitative Results:

**Figure 7:**
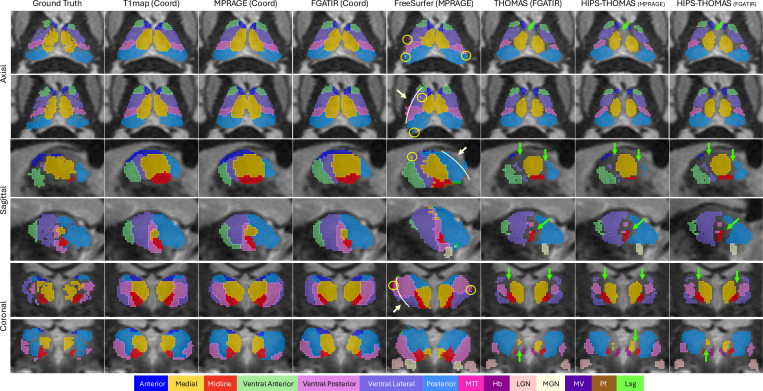
Qualitative comparison of thalamic nuclei segmentation performance against benchmark methods. Columns represent the manual ground truth, our three coordinate-aware CATNUS variants (T1 map, MPRAGE, and FGATIR), and three established methods including FreeSurfer (MPRAGE), THOMAS (FGATIR), and HIPS-THOMAS (MPRAGE and FGATIR). Rows show representative axial, sagittal, and coronal slices, each from a different subject. All predictions are overlaid on FGATIR images for visualization. The visualized segmentations include the seven major thalamic nuclear groups evaluated in this study, as well as additional nuclei (e.g., MGN, LGN, MTT, Hb, Pf, Lsg, MV) that appear in some benchmark outputs but are not labeled in the manual ground truth. Our CATNUS variants closely match manual annotations, producing smooth boundaries and anatomically coherent shapes. In contrast, FreeSurfer frequently generates fragmented predictions (yellow circles) and over-segments into adjacent structures beyond thalamic boundaries (white arrows). THOMAS and HIPS-THOMAS generate largely similar segmentations, yielding smoother results with fewer scattered errors than FreeSurfer, but tends to under-segment the thalamus, leaving some regions unlabeled (green arrows).

In Figure 7, we show segmentation outputs from CATNUS compared with FreeSurfer, THOMAS, and HIPS-THOMAS. All our coordinate-aware models produced segmentations that closely align with the manual ground-truth, exhibiting smooth boundaries, accurate localization for nuclear groups, and clear delineation of thalamic borders across all views. These segmentations successfully preserve internal thalamic structure and avoid spillage into surrounding non-thalamic regions, reflecting high spatial precision. In contrast, FreeSurfer frequently produced fragmented or noisy outputs, with scattered false positives marked by yellow circles. It also tends to over-segment the thalamus, spilling into adjacent subcortical regions beyond the thalamus boundary, as indicated by white arrows. THOMAS and HIPS-THOMAS generally produce very similar results. Compared with FreeSurfer, both methods yield visually smoother and more anatomically coherent segmentations, with improved nuclear localization and fewer isolated errors. However, they systematically under-segment the thalamus, leaving many regions unlabeled, as highlighted by green arrows. Overall, these qualitative observations reinforce our quantitative results, demonstrating that our approaches produce anatomically accurate and reliable segmentations that outperform existing benchmark methods.

#### Test-retest Analysis:

To evaluate segmentation robustness, we conducted a test-retest analysis on 10 healthy subjects from the mTBI study who underwent two MRI sessions (v1 and v2) six months apart. For each subject, the v2 data were rigidly co-registered to the corresponding v1 scan and processed using the pipeline described in Section 2.3. Each method was applied to both sessions, and the segmentation results were mapped into seven major nuclear groups. We computed the Dice similarity between v1 and v2 segmentations for each nuclear group and derived the VWA Dice score per subject. The final results in Table 1 show the mean and standard deviation (reported as percentages) across subjects. All three coordinate-aware models, particularly those trained on T1 map and FGATIR, achieved consistently high test-retest Dice scores, outperforming FreeSurfer, THOMAS and HIPS-THOMAS in nearly all groups. These findings indicate that CATNUS is not only accurate, but also robust and reproducible across repeated scans.

## 3.4 Out-of-Distribution Testing

### Experimental Design:

**Figure 8:**
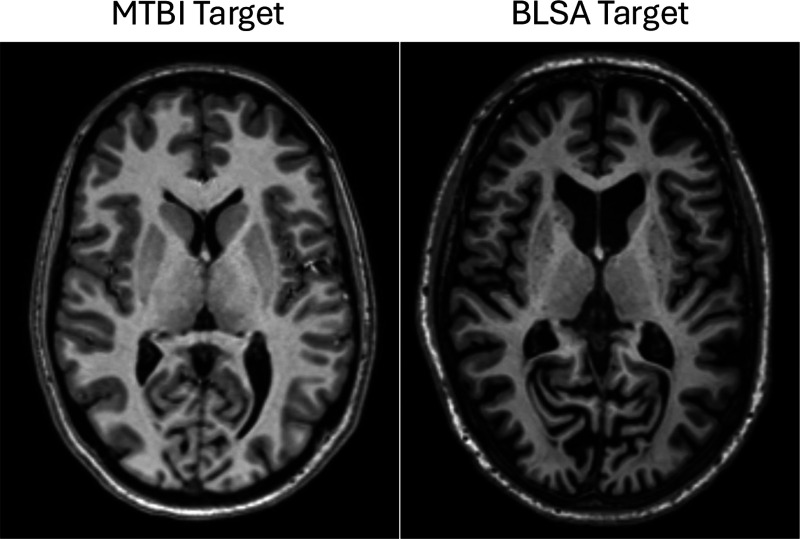
MTBI and BLSA harmonization targets used in OOD evaluation. Both are 3 T T1-weighted images shown on the same slice after identical preprocessing (registration to MNI152, N4 bias correction, and white-matter mean normalization). The MTBI target depicts a healthy young adult from the mild traumatic brain injury study (SIEMENS Prisma), whereas the BLSA target depicts a healthy aging participant from the Baltimore Longitudinal Study of Aging (Philips Achieva).

To evaluate model generalizability beyond the training domain, we performed out-of-distribution (OOD) testing using traveling-subject datasets. These datasets scan the same individuals across multiple sites, allowing quantitative assessment of segmentation stability under scanner and protocol variation, eliminating inter-subject differences as a confound. We used two publicly available traveling-subject datasets. The Frequently Traveling Human Phantom (FTHP) dataset [50] provides T1-weighted scans of a single healthy male (about 50 years old) acquired on 116 scanners over 2.5 years. For this analysis, 80 scans were used, consisting of four T1-weighted scans each from 20 different scanners (10 at 3.0T and 10 at 1.5T). The Multisite, Multiscanner, and Multisubject Acquisitions for Studying Variability (MASiVar) dataset [51] contains both diffusion and T1-weighted scans. We used only T1-weighted scans from cohort II, comprising five healthy adults (3 males, 2 females, ages 27–47), each scanned on four scanners with one to two sessions per scanner. Scanner details of both datasets are summarized in Table 2. All scans underwent identical preprocessing, including registration to MNI152 space, N4 bias field correction, and white-matter mean normalization, without joint processing with FGATIR. To systematically examine the impact of scanner- and protocol-dependent intensity differences on segmentation stability, we tested three input conditions: (1) Non-harmonized, preprocessed images reflecting inherent robustness to domain shift; (2) Harmonized-to-MTBI, images harmonized to the training domain (MTBI dataset) using Harmonization with Attention-based Contrast, Anatomy, and Artifact Awareness (HACA3) [52], assessing whether stability can be recovered when input contrast matches the training distribution; (3) Harmonized-to-BLSA, images harmonized to an independent domain from the Baltimore Longitudinal Study of Aging (BLSA) [53], reducing the possibility that improvements in (2) arise from the model recognizing familiar training patterns. The MTBI and BLSA targets differ in scanner vendor, acquisition protocol, and participant demographics, shown in Figure 8, avoiding confusion that stability improvements may stem from target similarity.

### Evaluation Strategy:

Because these datasets lack manual annotations, evaluation focused on two main aspects: (1) quantitative stability, measured by the coefficient of variation (CV = standard deviation/mean volume) across repeated scans of the same subject, and (2) qualitative consistency, assessed by visual inspection of segmentation plausibility and anatomical coherence across scanners. For FTHP, CVs were computed across all 80 scans to measure scanner-induced variability. For MASiVar, subject-level CVs were first computed across scanners and then averaged across five subjects. We compared CATNUS against FreeSurfer and HIPS-THOMAS. HIPS-THOMAS was chosen instead of THOMAS because it is optimized for T1-weighted MRI and provides more accurate and reliable segmentations for for conventional MPRAGE scans. Outputs from all methods were mapped to seven major nuclear groups according to the protocol in Figure 3 for comparison.

### Quantitative Results:

Table 3 reports the CV across all methods and harmonization conditions. On the FTHP dataset (Table 3a-c), non-harmonized data showed FreeSurfer achieving the lowest overall CV at 4.0%, followed by CATNUS at 6.2% and HIPS-THOMAS at 6.7%. However, harmonization altered this ranking. CATNUS improved nearly six-fold to 1.1% for both MTBI and BLSA targets, outperforming FreeSurfer (1.9% MTBI; 1.8% BLSA) and HIPS-THOMAS (6.5% for both). On the MASiVar dataset (Table 3d-f), non-harmonized HIPS-THOMAS achieved the lowest overall CV (1.8%) compared to CATNUS (5.8%) and FreeSurfer (7.0%). After harmonization, CATNUS again achieved the best stability, with overall CVs of 0.8% (MTBI) and 1.1% (BLSA), surpassing HIPS-THOMAS (1.6% MTBI; 1.5% BLSA) and FreeSurfer (3.9% MTBI; 2.4% BLSA). Across major nuclear groups, both datasets showed the same pattern: harmonization consistently reduced variability for CATNUS and FreeSurfer, with CATNUS showing consistently low CVs after harmonization, while HIPS-THOMAS remained largely unchanged. In addition, performance was nearly identical under MTBI- and BLSA-harmonization (e.g., overall CVs of 1.1% vs 1.1% on FTHP, and 0.8% vs 1.1% on MASiVar), suggesting stability gains stem from intensity harmonization itself rather than alignment to a specific training domain.

### Qualitative Results:

**Figure 9:**
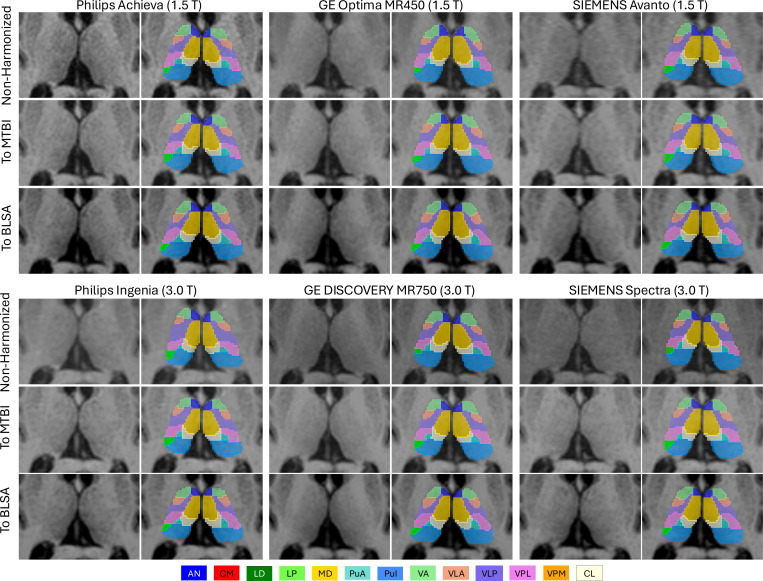
Qualitative results of out-of-distribution (OOD) segmentation on the FTHP traveling-subject dataset. Results are shown for six scanners spanning 1.5 T and 3.0 T field strengths from three vendors (Philips, GE, SIEMENS). All scans were acquired from the same subject, and the same anatomical slice is shown after registration to the MNI space. Rows correspond to three harmonization conditions: non-harmonized, harmonized to MTBI, and harmonized to BLSA.

**Figure 10:**
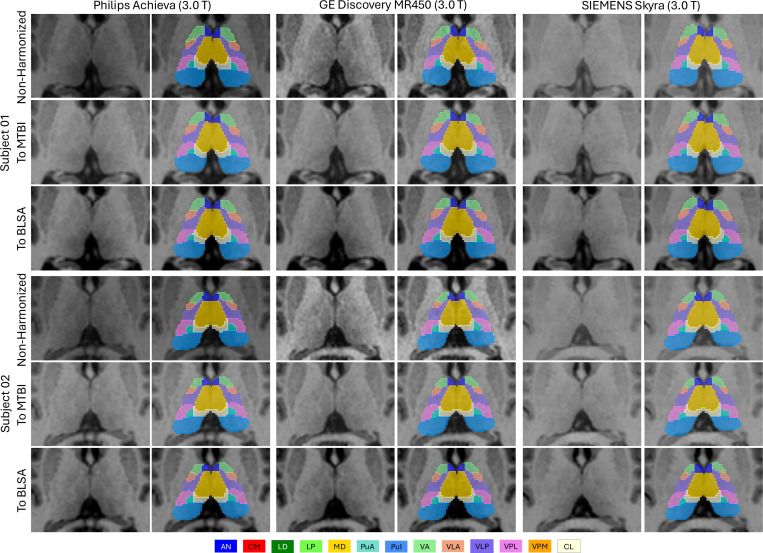
Qualitative results of out-of-distribution (OOD) segmentation on the MASiVar traveling-subject dataset. Results are shown for two representative subjects scanned on three scanners (Philips Achieva, GE Discovery MR450, and SIEMENS Skyra). For each subject and scanner, the same anatomical slice after registration to the MNI space is shown, with predicted thalamic nuclei overlaid. Rows correspond to three harmonization conditions: non-harmonized, harmonized to MTBI, and harmonized to BLSA T1-weighted data.

Visual inspection confirmed strong generalization across scanners and sites. Figure 9 shows representative segmentations from the FTHP dataset across six scanners spanning 1.5 T and 3.0 T field strengths and three major vendors (Philips, GE, and SIEMENS). Despite being trained only on 3.0 T data, our model consistently localized the thalamus and nuclei with smooth, anatomically coherent boundaries across all scanners. Figure 10 shows segmentations from the MASiVar dataset for two subjects scanned on three different scanners (Philips, GE, and SIEMENS). Our model accurately captured inter-subject anatomical differences, with thalamic size, nuclear proportions, and shapes varying between individuals. Within each subject, segmentations remained stable across scanners. The same anatomical structures maintained consistent boundaries, small nuclei preserved their positions, and spatial relationships between nuclear groups stayed coherent. In addition, the model produced anatomically faithful segmentations even on non-harmonized data, indicating strong inherent generalization, with harmonization further improving stability across scanners.

## 4 Discussion

In the following section, we discuss several key insights derived from our experiments. First, we find that among MPRAGE, FGATIR, and quantitative T1 maps, the T1 maps consistently yield superior segmentation performance across thalamic nuclei, aligning with the findings in our prior study [44]. This advantage can be explained from the perspective of MRI signal formation. The intensity of a T1-weighted image is governed by two intrinsic tissue properties: the longitudinal relaxation time (T1) and proton density (PD). Given these two parameters, one can mathematically synthesize any T1-weighted image, including MPRAGE and FGATIR, by adjusting the inversion and repetition times. In practice, however, PD maps contribute little unique contrast beyond what is already encoded in the T1 maps [44]. As a result, the important tissue-specific contrast across different T1-weighted acquisitions is inherently captured in the T1 maps. Although certain features in the T1 maps may not be visually apparent to human raters, deep learning models are capable of leveraging subtle patterns and contextual cues to delineate complex anatomical structures, which may explain why models trained on T1 maps alone can achieve high segmentation accuracy. That said, should we still compute multi-TI images from the T1 maps? We argue yes, but for a different purpose. While unnecessary for training segmentation models, multi-TI images offer enhanced visual contrast that helps human raters in identifying thalamic boundaries and selecting optimal TIs for manual annotation. Thus, T1 maps are sufficient for model input, while multi-TI images remain valuable for human-guided delineation and quality control. One might question why we chose single-input models rather than multi-channel inputs. While theoretically we could use MPRAGE, FGATIR, and T1 maps as multi-channel inputs, we chose a single-input strategy for the following reasons: First, not all clinical environments can simultaneously acquire multiple T1-weighted sequences. Second, when MPRAGE and FGATIR are available, T1 maps can be computed from these acquisitions, and T1 maps inherently encode the core tissue contrast information needed for segmentation. Additional input channels would introduce feature redundancy, potentially dispersing the model’s learning capacity and increasing computational complexity. Therefore, based on efficiency and practicality principles, we provide three independent single-input models for different clinical scenarios.

Our second key finding is that coordinate convolution(CoordConv) greatly improves segmentatio n performance for small nuclei, showing minimal effect on larger structures. In our experiments, the anterior pulvinar (PuA) nucleus, the smallest thalamic structure, frequently missed or mislocalized without coordinate encoding, but was segmented accurately when CoordConv was used. This improvement stems from the inherent difficulty of segmenting small anatomical regions. Small nuclei occupy very few voxels, are often underrepresented in training, and are more susceptible to class imbalance. In encoder–decoder architectures like U-Net, higher-level features tend to emphasize large, salient regions, making small structures easier to overlook [54]. In addition, small nuclei often have complex, irregular boundaries and are more sensitive to spatial perturbations introduced by data augmentation, interpolation, and pooling, leading to unstable predictions and noisy edges. CoordConv mitigates these issues by explicitly providing the network with normalized spatial coordinates, giving it direct access to positional information [45]. This reduces the burden of inferring spatial structure from appearance and enables the model to learn location-aware features more efficiently. With CoordConv, the network can treat spatial position as a continuous and stable signal, leading to smoother anatomical boundaries and more consistent predictions.

As a third analysis, we compared CATNUS against three widely used thalamic segmentation tools: FreeSurfer, THOMAS and HIPS-THOMAS. Each method was applied to its recommended input modality (MPRAGE for FreeSurfer, FGATIR for THOMAS, both MPRAGE and FGATIR for HIPS-THOMAS), and all outputs were mapped to a unified labeling scheme. CATNUS consistently outperformed these baselines in segmentation accuracy and test–retest reliability across all major thalamic regions. It is also more efficient, producing a full 3D segmentation in under one minute on a single GPU, compared to approximately 10 minutes for THOMAS and HIPS-THOMAS, and several hours for FreeSurfer. However, we must acknowledge a potential source of bias in this comparison. CATNUS was trained and evaluated using manual annotations specifically created for this study, while FreeSurfer, THOMAS and HIPS-THOMAS were developed based on different atlases and labeling conventions. Therefore, our model may be more closely aligned with the evaluation labels, which could artificially favor its performance.

As a fourth analysis, we evaluated the out-of-distribution (OOD) generalizability of CATNUS using traveling-subject datasets, which isolate scanner-induced variability while keeping anatomy constant. Results showed that CATNUS produced anatomically coherent segmentations even without harmonization, demonstrating inherent generalization to unseen domain. Harmonization further improved cross scanner consistency and reduced volumetric variability across repeated scans. Therefore, during inference, harmonization is applied as a preprocessing step prior to segmentation to ensure robust performance. Different methods benefited from harmonization to varying degrees. CATNUS and FreeSurfer both showed improved stability, with CATNUS exhibiting a larger gain due to its higher sensitivity to input intensity distribution. FreeSurfer, while more robust to contrast variations owing to its probabilistic atlas framework, also achieved better consistency after harmonization. In contrast, HIPS-THOMAS showed little improvement, as its polynomial synthesis model already performs an implicit form of intensity normalization. We also found that harmonization toward different targets (MTBI vs. BLSA) yielded comparable results, suggesting that the observed improvements stem from consistent intensity statistics rather than matching to the training domain. However, the current CATNUS framework supports harmonization only for standard T1-weighted images using HACA3. We are exploring extensions to T1 maps and FGATIR, which may further enhance the model’s robustness across supported input modalities.

In addition to the limitations discussed above, our study has several others that warrant consideration. First, the training dataset is relatively small, withonlyaround20subjects, limited by the high cost and complexity of obtaining accurate manual delineations of thalamic nuclei. In addition, all training subjects were young adults from a single clinical cohort (mTBI study), which limits anatomical diversity. Second, the manual annotations are sparse, with only high-confidence voxels labeled. While this improves label precision, it also leaves many thalamic voxels unused during training, reducing the amount of available supervision. Third, our evaluation is based only on the true positive rate, which measures sensitivity on labeled voxels but does not penalize false positives in unlabeled regions. Fourth, the current labeling protocol does not include several important thalamic structures, such as the medial and lateral geniculate nuclei (MGN and LGN). Their absence limits the utility of our method in certain studies. Fifth, our T1 maps were computed from a dual inversion time approach. However, T1 quantification can be performed using various alternative methods, including MP2RAGE [55], variable flip angle [56], and multi-point inversion recovery sequence [57], which may produce T1 maps with different contrast characteristics and numerical ranges. Model performance on T1 maps derived from these methods remains to be validated. Finally, OOD testing was conducted only on standard T1-weighted images because no public datasets currently provide T1 map or FGATIR acquisitions. Future work will focus on addressing these limitations and expanding the applicability of CATNUS across modalities and populations, as well as exploring its clinical applications in disease cohorts for detecting structural changes and informing treatment planning.

## 5 Conclusion

In this work, we presented CATNUS, a deep learning–based framework for segmenting 13 thalamic nuclei (or nuclear groups) from T1-weighted MRI using a coordinate-aware 3D U-Net. By incorporating voxel-wise spatial coordinates, CATNUS improves anatomical localization, particularly for small and low-contrast nuclei. To support broad clinical applicability, we provided pretrained model variants that operate on quantitative T1 maps, MPRAGE, or FGATIR scans, for flexible deployment. Compared with FreeSurfer, THOMAS, and HIPS-THOMAS, CATNUS achieved higher segmentation accuracy and test–retest reliability across multiple nuclei. The model also demonstrated strong generalization to out-of-distribution datasets, producing stable and anatomically coherent segmentations across scanners, field strengths, and vendors. Overall, CATNUS delivers an accurate, generalizable, and clinically accessible solution for thalamic nuclei segmentation, offering strong potential to advance both neuroimaging research and clinical assessment.

## Figures and Tables

**Table 1:** Test-retest Dice scores for CATNUS against FreeSurfer, THOMAS, and HIPS-THOMAS. Results are computed on 10 healthy subjects across seven major nuclear groups and volume-weighted average (VWA), shown in %. The highest value each row is shown in bold.

Nuclear Group	T1map (Coord)	MPRAGE (Coord)	FGATIR (Coord)	FreeSurfer (MPRAGE)	THOMAS (FGATIR)	HIPS-THOMAS (MPRAGE)	HIPS-THOMAS (FGATIR)
Anterior	**85.42±12.02**	84.35±8.95	82.85±12.48	75.43±10.53	80.62±11.76	79.34±11.86	81.10±11.42
Medial	**93.48±6.05**	93.06±5.65	91.77±7.13	87.57±5.21	89.72±5.60	88.96±6.39	89.86±6.11
Midline	**89.28±8.56**	85.95±10.40	88.39±7.23	80.97±7.07	82.97±9.71	83.00±11.04	82.95±10.38
Ventral Anterior	**89.96±8.27**	88.15±7.94	88.77±7.85	82.32±6.08	84.97±8.53	84.20±9.46	84.98±8.59
Ventral Posterior	88.29±7.22	84.69±10.03	**88.85±8.39**	83.70±6.64	85.48±7.07	81.47±6.40	85.37±7.16
Ventral Lateral	91.67±6.49	91.00±5.65	**92.35±6.12**	86.47±5.43	89.39±5.42	87.88±6.39	89.70±5.45
Posterior	93.05±4.24	91.87±4.33	**93.44±5.05**	88.67±4.48	90.90±3.54	91.29±3.74	90.96±3.67

VWA	**91.65±6.17**	90.36±6.12	91.31±6.59	86.14±5.06	88.90±5.35	88.06±5.81	89.02±5.54

**Table 2:** Scanner Information, including vendor, model, and field strength (FS) for three datasets. The MTBI dataset was used for training, while the FTHP and MASiVar are traveling-subject datasets for out-of-distribution (OOD) testing. In MASiVar, Site V includes two Philips Achieva scanners, denoted as ‘Achieva’ and ‘Achieva*’.

Dataset	Site	Vendor	Model	FS
MTBI	Site A	SIEMENS	Prisma	3.0T

FTHP	Site B	Philips	Intera	1.5T
	Site C	SIEMENS	Aera	1.5T
	Site D	SIEMENS	Avanto	1.5T
	Site E	Philips	Achieva	1.5T
	Site F	SIEMENS	Espree	1.5T
	Site G	SIEMENS	Symphony	1.5T
	Site H	GE	Optima MR450w	1.5T
	Site I	Philips	Ingenia	1.5T
	Site J	GE	OPTIMA MR360	1.5T
	Site K	GE	Signa HDxt	1.5T
	Site L	GE	DISCOVERY MR750	3.0T
	Site M	SIEMENS	Skyra	3.0T
	Site N	Philips	Ingenia	3.0T
	Site O	SIEMENS	Verio	3.0T
	Site P	Philips	Achieva	3.0T
	Site Q	SIEMENS	MAGNETOM Vida	3.0T
	Site R	SIEMENS	Spectra	3.0T
	Site S	SIEMENS	MAGNETOM Lumina	3.0T
	Site T	GE	SIGNA Architect	3.0T
	Site U	SIEMENS	Prisma	3.0T

MASiVar	Site V	Philips	Achieva	3.0T
	Site V	Philips	Achieva*	3.0T
	Site W	GE	Discovery MR750	3.0T
	Site X	SIEMENS	Skyra	3.0T

**Table 3:** Quantitative results of out-of-distribution (OOD) segmentation using traveling-subject datasets. We assess CATNUS generalizability on the FTHP and MASiVar datasets under three harmonization conditions: non-harmonized, harmonized to MTBI, and harmonized to BLSA. Segmentation stability was quantified using the coefficient of variation (CV = standard deviation / mean volume), reported in %. Lower CV values indicate greater segmentation stability and are highlighted in bold. “Overall” represents the combined thalamic volume across all nuclear groups.

(a) FTHP - NON-Harmonized Data								

CV (Std/Mean)	Anterior	Medial	Midline	Ventral Anterior	Ventral Posterior	Ventral Lateral	Posterior	Overall

CATNUS	15.8%	10.3%	9.3%	14.5%	13.1%	7.8%	6.9%	6.2%
HIPS-THOMAS	17.9%	9.2%	10.2%	7.0%	8.2%	7.2%	7.4%	6.7%
FreeSurfer	**11.2%**	**7.6%**	**4.9%**	**6.5%**	**5.5%**	**4.2%**	**6.6%**	**4.0%**

(b) FTHP - Harmonized Data to MTBI								

CV (Sth/Mean)	Anterior	Medial	Midline	Ventral Anterior	Ventral Posterior	Ventral Lateral	Posterior	Overall

CATNUS	**2.7%**	**2.0%**	**2.4%**	**3.3%**	**2.7%**	**1.8%**	**1.3%**	**1.1%**
HIPS-THOMAS	16.7%	8.7%	10.2%	6.8%	7.2%	7.0%	7.0%	6.5%
FreeSurfer	7.1%	3.8%	3.5%	3.8%	3.3%	2.2%	3.7%	1.9%

(c) FTHP - Harmonized Data to BLSA								

CV (Std/Mean)	Anterior	Medial	Midline	Ventral Anterior	Ventral Posterior	Ventral Lateral	Posterior	Overall

CATNUS	**3.0%**	**2.0%**	**2.0%**	3.9%	**2.4%**	**1.6%**	**1.3%**	**1.1%**
HIPS-THOMAS	18.3%	8.2%	9.4%	6.6%	7.4%	6.9%	6.9%	6.5%
FreeSurfer	6.4%	3.6%	3.6%	**3.3%**	3.3%	2.3%	3.4%	1.8%

(d) MASiVar – NON-Harmonized Data								

CV (Std/Mean)	Anterior	Medial	Midline	Ventral Anterior	Ventral Posterior	Ventral Lateral	Posterior	Overall

CATNUS	**5.1%**	7.2%	12.9%	8.2%	15.0%	11.6%	12.2%	5.8%
HIPS-THOMAS	7.6%	**2.9%**	**5.3%**	**2.4%**	**5.7%**	**2.5%**	**1.6%**	**1.8%**
FreeSurfer	7.5%	6.6%	10.0%	5.0%	14.3%	9.7%	3.9%	7.0%

(e) MASiVar - Harmonized Data to MTBI								

CV (Std/Mean)	Anterior	Medial	Midline	Ventral Anterior	Ventral Posterior	Ventral Lateral	Posterior	Overall

CATNUS	**3.2%**	**2.2%**	**2.4%**	**2.0%**	**1.6%**	**2.2%**	**1.1%**	**0.8%**
HIPS-THOMAS	6.3%	3.0%	7.0%	3.0%	4.4%	2.3%	2.2%	1.6%
FreeSurfer	6.8%	2.7%	4.8%	2.8%	8.1%	4.4%	3.8%	3.9%

(f) MASiVar - Harmonized Data to BLSA								

CV (Std/Mean)	Anterior	Medial	Midline	Ventral Anterior	Ventral Posterior	Ventral Lateral	Posterior	Overall

CATNUS	**3.6%**	**2.5%**	**2.2%**	**2.5%**	**1.7%**	**1.9%**	**1.2%**	**1.1%**
HIPS-THOMAS	5.5%	3.3%	5.9%	3.2%	4.8%	**1.9%**	2.1%	1.5%
FreeSurfer	7.4%	2.6%	3.2%	2.7%	5.6%	3.0%	2.8%	2.4%

## Data Availability

The MRI data used in this study were obtained from multiple sources, including our institutional MRI unit and publicly available datasets. The MTBI dataset, collected under local research ethics committee approval, is not publicly available due to participant privacy and regulatory restrictions. All remaining data used in this work are publicly accessible: the FTHP dataset (https://www.nitrc.org/projects/fthp/), the MASiVar dataset (https://openneuro.org/datasets/ds003416/), and the BLSA dataset (https://www.blsa.nih.gov/).

## Code Availability Statement

The pretrained models and codes will be publicly available upon acceptance. The framework will be distributed as a Singularity container for easy deployment. The container supports T1 map, MPRAGE, or FGATIR inputs, automatically performs all preprocessing and segmentation steps, and outputs thalamic nuclei segmentations for each image.
